# Differences in Knee Shape between ACL Injured and Non-Injured: A Matched Case-Control Study of 168 Patients

**DOI:** 10.3390/jcm10050968

**Published:** 2021-03-02

**Authors:** Koen S.R. van Kuijk, Vincent Eggerding, Max Reijman, Belle L. van Meer, Sita M.A. Bierma-Zeinstra, Ewoud van Arkel, Jan H. Waarsing, Duncan E. Meuffels

**Affiliations:** 1 Department of Orthopedic Surgery, Erasmus MC University Medical Centre, 3015 CN Rotterdam, The Netherlands; v.eggerding@erasmusmc.nl (V.E.); m.reijman@erasmusmc.nl (M.R.); s.bierma-zeinstra@erasmusmc.nl (S.M.A.B.-Z.); e.waarsing@erasmusmc.nl (J.H.W.); d.meuffels@erasmusmc.nl (D.E.M.); 2 Department of Radiology, Albert Schweitzer hospital, 3318 AT Dordrecht, The Netherlands; 3 Department of Sports Medicine, St Antonius Hospital, 3543 AZ Utrecht, The Netherlands; bellevanmeer@hotmail.com; 4 Department of General Practice, Erasmus MC University Medical Centre, 3015 CN Rotterdam, The Netherlands; 5 Department of Orthopaedic Surgery, Haaglanden Medical Centre Haaglanden, 2512 VA The Hague, The Netherlands; e.van.arkel@haaglandenmc.nl

**Keywords:** ACL, femoral intercondylar notch, knee anatomy, ACL prevention, ACL risk factors

## Abstract

Objective: Anterior cruciate ligament (ACL) injury prevention programs could be more effective if we could select patients at risk for sustaining an ACL rupture. The purpose of this study is to identify radiographic shape variants of the knee between patients with and patients without an ACL rupture. Methods: We compared the lateral and Rosenberg view X-rays of 168 prospectively followed patients with a ruptured ACL to a control group with intact ACLs, matched for gender, after knee trauma. We used statistical shape modeling software to examine knee shape and find differences in shape variants between both groups. Results: In the Rosenberg view X-rays, we found five shape variants to be significantly different between patients with an ACL rupture and patients with an intact ACL but with knee trauma. Overall, patients who had ruptured their ACL had smaller, flatter intercondylar notches, a lower lateral tibia plateau, a lower medial spike of the eminence, and a smaller tibial eminence compared to control patients. Conclusion: Patients with an ACL rupture have smaller intercondylar notches and smaller tibial eminences in comparison to patients with an intact ACL after knee trauma.

## 1. Introduction

A rupture of the anterior cruciate ligament (ACL) is a common, usually sports-related injury. The annual incidence varies between 5 and 8 per 10,000 persons in the Western population [1,2,3,4]. Rupture of the ACL has immediate consequences resulting in swelling of the knee and pain, but also long-term consequences, as there is an almost fourfold risk to progress to moderate or severe radiological osteoarthritis after ten years [5]. Furthermore, in the young population, ACL rupture has a direct impact on sport participation. It has been found, for instance, that after ACL reconstruction, 82% of the patients returned to sport participation; however, only 63% returned to their preinjury sport level [6,7]. Amongst young patients who return to their pre-trauma sports activity, the prevalence of a re-rupture of their ACL may be as high as 30% [8,9]. Additionally, reports show that around 7% of patients need revision ACL surgery and around 3.4% of patients have ACL reconstructions on the contralateral side [10].

This has led to a rise in interest in the mechanism of ACL rupture, in risk factors, prevention of ACL rupture and secondary ACL injury. Neuromuscular and proprioceptive prevention programs have been demonstrated to significantly reduce the prevalence of ACL ruptures in young athletes by approximately 50% [11,12,13,14]. However, these prevention programs can be more efficient if they focus on athletes who are at increased risk of sustaining an ACL rupture. Therefore, it is essential to understand the mechanisms that lead to ACL rupture and to identify individuals with an increased risk of ACL rupture.

There is a relationship between shape variants of the knee and the need for reconstruction of the ACL after rupture [15]. This has encouraged us to investigate the relationship between shape and rupture of the ACL more profoundly. Risk factors for ACL rupture can be categorized into anatomical, hormonal, neuro-mechanical, and environmental. In the present study, we focused on osseous anatomical risk factors; anatomical risk factors have previously been studied with a focus on selected aspects of the anatomical properties of the knee. Anatomical factors that have previously been reported to be related to the risk of ACL rupture are increased tibial slope, decreased femoral notch size, and smaller ACL size [16,17]. With the use of statistical shape modeling (SSM), a hypothesis generating-methodology that identifies independent shape variants, we can quantitatively describe the complete morphology of a bone or joint. SSM reproduces all variation in shape that is present in the studied population. With the use of SSM, we can identify new shape variants of the knee that are clinically relevant in relation to an ACL rupture. Furthermore, it enables us to objectively review shape variants that have been investigated before. Although not all clinicians will have a program such as SSM in use, the results of this study can be used in daily practice and can help doctors in selecting patients at greater risk for sustaining an ACL rupture.

SSM has been used earlier by our group to determine whether certain shape aspects are correlated to clinical outcomes after ACL rupture [18]. We found that operatively treated patients with good subjective outcomes had a smaller intercondylar notch and a lower width intercondylar eminence, as evaluated by The International Knee Documentation Committee (IKDC) questionnaire, compared to patients with worse outcome. Non-operatively treated patients with good subjective outcomes had a more pyramidal shaped intercondylar notch.

The purpose of this study is to find radiographic shape variants of the knee between patients with and patients without an ACL rupture, which can be used in daily practice to help select patients with a greater risk for sustaining an ACL rupture.

## 2. Experimental Section

### 2.1. Cases

We included patients with a ruptured ACL from two previous series: the KNALL [19] and the CAS-ACL study [20].

The KNee osteoArthritis anterior cruciate Ligament Lesion (KNALL) study is a prospective observational follow-up study of 154 patients with a recent ACL rupture, who were treated operatively or non-operatively. Patients were selected from January 2009 to November 2010, and there was a two-year follow-up period. Physical examination and MRI confirmed ACL rupture. Patients were included from three collaborating hospitals.

The CAS-ACL study is a double-blinded randomized controlled trial of 100 patients who underwent ACL reconstruction. In this study, computer-assisted ACL reconstruction was compared to conventional ACL reconstruction [20,21]. The inclusion period was from January 2007 to November 2009 with a two-year follow-up period. Of the 254 patients included in the two studies, 183 had both Rosenberg view and lateral view radiographs and were enrolled in the present study.

All patients (both ACL injured as healthy controls) included had a Kellgren and Lawrence grade 0–1 at presentation (no radiological signs of osteoarthritis). Our medical ethics committee (MEC-2006-223 and MEC-2008-068) approved both studies. For the use of the data of control patients, the medical ethics committee ruled that no specific approval was required (MEC-2017-422).

### 2.2. Controls

**Figure 1:**
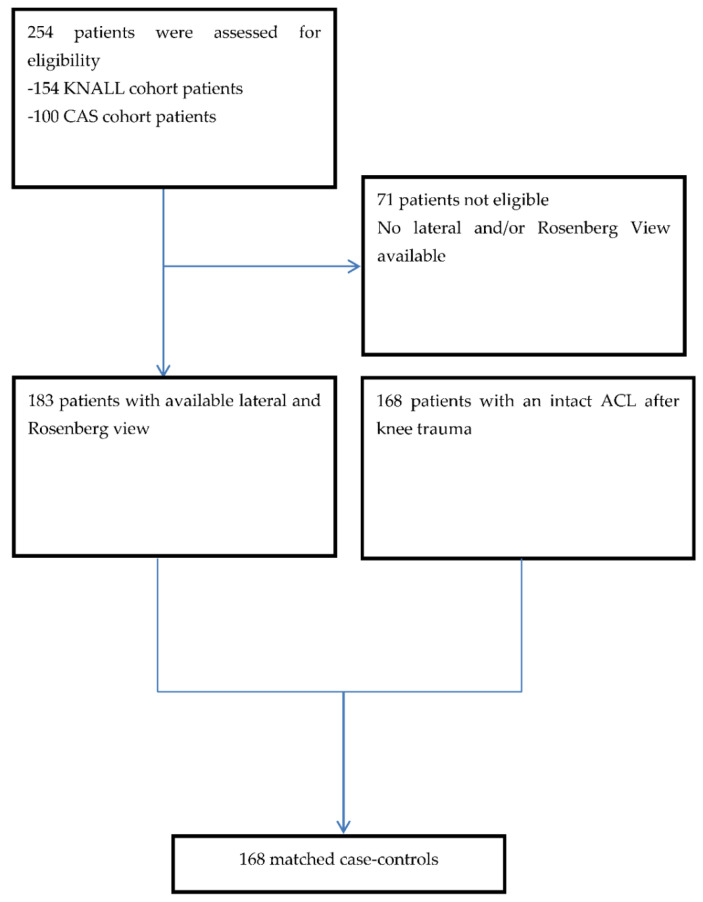
Flowchart of the selected patients included in the study. KNee osteoArthritis anterior cruciate Ligament Lesion (KNALL); Computer Assisted Surgery (CAS).

The control group consisted of patients identified retrospectively from the hospital records. They had consulted a trauma or orthopedic surgeon because of a knee trauma (median of 3 months and a range of 1–60 months between trauma and X-ray) with confirmed intact ACL by MRI and/or arthroscopy. Hospital records from January 2003 until July 2013 were searched. Patients were selected for the control group if they had both standard lateral view and Rosenberg view radiographs at the time of the first consult, were practicing sports before the injury, and had a Kellgren and Lawrence grade 0–1 at presentation (no radiological signs of osteoarthritis). Control patients and cases were matched for gender. For age, our patients were matched with a control patient older in age. We chose older control subjects to ensure that the older controls were exposed to pivoting sports for a longer period. This way, they were more sufficiently at risk for sustaining an ACL rupture. Of all patients found in the database, 168 control patients were matched to 168 patients with a ruptured ACL. See Figure 1. We were unable to match all patients due to younger age, since we wanted to only include older control patients. Fifteen control patients were younger than the matches from the ACL ruptured group.

### 2.3. X-rays and Statistical Shape Modeling

We performed the radiological measurements using standard lateral view X-rays and Rosenberg view X-rays. The Rosenberg view is a weight-bearing postero-anterior radiograph taken at 45° flexion of the knee [22]. We chose to include the Rosenberg view X-rays because it gives a better view of the intercondylar notch and gives better insights in the shape of the femur.

With statistical shape modeling (SSM) [23], it is possible to quantify all shape aspects of the knee joint in the radiographs. This method is unique because it dissects nearly all possible shape variations into a limited number of objectively quantitative measures that each describe a certain shape variant. SSM has been used in studies of a possible association between knee shape variants and osteoarthritis [24]. In the radiographs, we outlined the distal femur, the proximal tibia, and fibula (ASM tool kit, Manchester University, Manchester, UK).

For the lateral view X-rays, the femur and tibia were outlined by 60 landmark points on the bones. For the Rosenberg view, 25 landmark points were necessary to completely outline the bones. Each point was placed in the same location in each image, as precisely as possible, to allow a comparison between shapes. For the exact placement of each landmark point, see the addendum. Statistical shape modeling transforms the set of points into a statistical shape model, which comprises a number of shape variants that together explain 95% of the variation in the shape of the individual knee of the study population. SSM represents relative variation in shape, independent of differences in the size of the joint. In this way, the method corrects errors caused by variation in magnification or in the size of the patient’s knee.

Intraobserver reliability was established by randomly selecting 25 Rosenberg view X-rays of patients with a ruptured ACL and 25 Rosenberg view X-rays of patients with an intact ACL, which were outlined a second time after 2 weeks.

The description of which shape aspects a variant represents was determined at a consensus meeting. At this consensus meeting, an orthopedic surgeon, an expert on SSM, the first authors, and the principal investigator determined the different shape variants.

### 2.4. Statistical Analysis

We used logistic regression analysis to study the association between each shape variant and whether or not patients had a ruptured ACL. As the dependent variable, we used whether or not a patient had an ACL rupture (yes or no), and as independent variables, we selected the different variants. We applied Bonferroni correction for multiple testing. We investigated if there was a significant effect of the X-ray protocol on knee shape by comparing the shape models of the X-rays taken in the three participating hospitals. Furthermore, we analyzed if correction for age changed the outcomes. All statistical analyses were performed with IBM SPSS Statistics for Windows (Version 20.0. IBM Corp., Armonk, NY, USA).

## 3. Results

### 3.1. Patients

The study population consisted of two groups of 168 patients; each group consisted of 119 males and 49 females. The mean age of the 168 patients after ACL rupture was 31 (±standard deviation (SD) 7.4) years and of the control group 38 (±SD 12) years (Table 1). The diagnoses of the included control patients can be found in Table 1, including additional injuries of the ACL ruptured patients. The mean time between trauma and radiograph was 1.0 months for the ACL injured and 6.9 months for the control group.

### 3.2. SSM

SSM produced 30 variants for the Rosenberg view and 24 variants for the lateral view X-rays. After we applied Bonferroni correction for multiple testing, we considered a *p*-value of 0.0017 for the Rosenberg view (0.05/30 = 0.00167) and a *p*-value of 0.0021 for the lateral view (0.05/24 = 0.0021) as statistically significant.

In the Rosenberg view, five variants were significantly associated with rupture of the ACL (see Table 2). For the lateral view X-rays, none of the variants were statistically significantly associated with rupture of the ACL. For every increase of 1 SD, the OR is given, meaning that if a patient scores 1 SD in a specific variant, the given OR is the odds ratio for sustaining an ACL rupture compared to a patient who scores the mean (0 SD).

We analyzed whether the protocols of the X-rays differed in the period of time of which the X-rays were taken. We did not find a significant difference between the three hospitals, nor did we find a significant difference in time. Correction for age did not alter the outcomes; therefore, we did not correct for age.

The intraobserver (ICC) was considered good to excellent with a range of 0.48–0.97 and 89% above 0.7.

### 3.3. Description of the Variants

**Figure 2:**
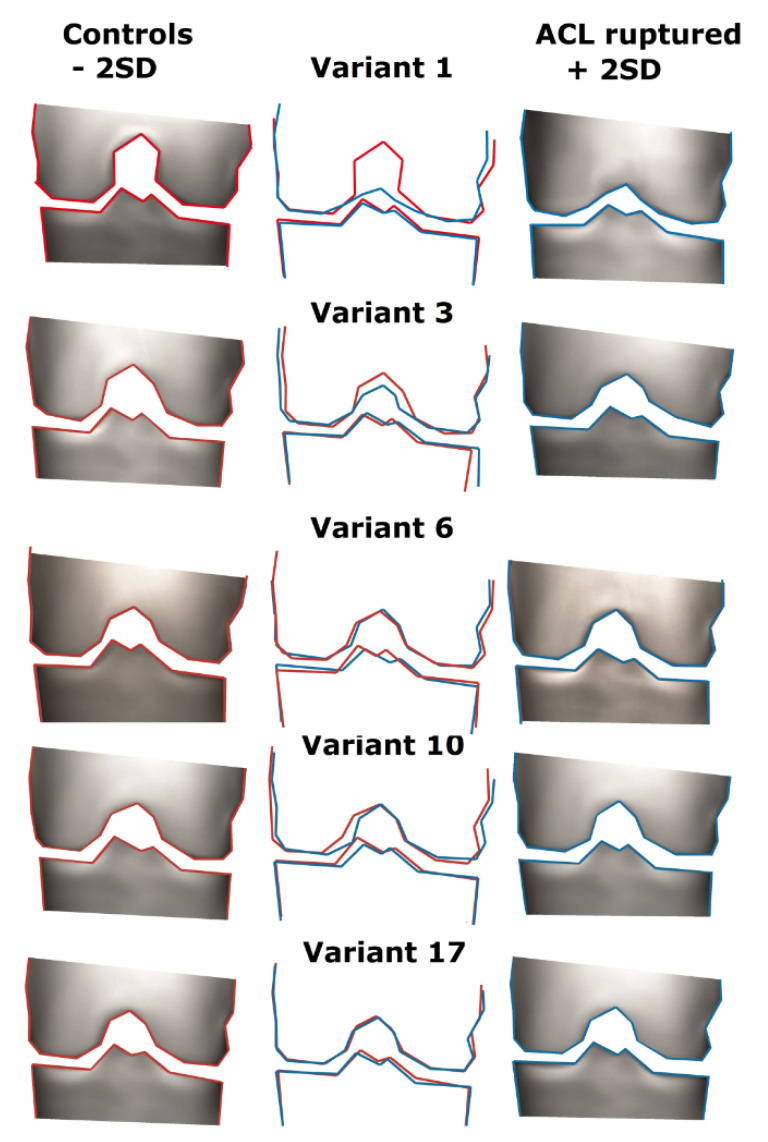
Graphic outcomes of statistic shape modeling: five variants that are significantly different for patients with intact and ruptured ACL. On the left and right sides are the two extremes (+/− 2.5 SD); in the middle is the overlay of both sides. SD = Standard.

Here, we present the description, defined at the consensus meeting, of the variants significantly associated with an ACL rupture. In Figure 2, we present the graphics of each variant. On the outside, the +2SD and -2SD variants are shown; in the middle, we present an overlay. Higher variants describe more subtle shape aspects, e.g., the variation in shape represented in shape variant 17 is much more subtle than the variation represented by shape variant 1.

Variant 1

Variant 1 describes a variation in the height of the intercondylar notch. Positive values represent a more flattened intercondylar notch. Patients with an ACL rupture had flatter intercondylar notches than control patients.

Variant 3

Variant 3 shows a variation in the width and height of the intercondylar notch. Positive values represent a smaller intercondylar notch. Patients with an ACL rupture had smaller intercondylar notches than control patients.

Variant 6

Variant 6 represents the size of the footprint of the ACL on the tibial eminence. Positive values represent a smaller, flatter tibial eminence. Patients with an ACL rupture had a smaller tibial eminence than control patients.

Variant 10

Variant 10 outlines the footprint of the ACL on the tibia, the width of the tibial eminence, and the width of the intercondylar notch. Positive values represent a smaller tibial eminence and a smaller intercondylar notch. Patients with ACL rupture had a smaller tibial eminence and a smaller intercondylar notch.

Variant 17

Variant 17 depicts a very subtle difference. Positive values represent a lower height of the lateral tibial plateau and the lower medial spike of the tibial eminence. Patients with an ACL rupture had a lower lateral tibia plateau and a lower medial spike of the intercondylar eminence.

## 4. Discussion

The most important finding of the present study is that aspects of bony morphology in the Rosenberg view X-ray of the knee joint were different between patients with a ruptured ACL and a matched control group. Our findings indicate that a smaller, flatter intercondylar notch; a lower lateral tibia plateau; a lower medial spike of the eminence; and a smaller tibial eminence were more common in patients who ruptured their ACL compared to control patients. Lower body strength exercises (for example, Nordic hamstring, lunges, and heel-calf raise) are not performed by all (professional) athletes but have been proven to reduce the risk of ACL rupture [25]. If we can identify patients at higher risk for ACL injury, injury prevention programs might be even more effective, although this should be confirmed in a different study. Our results could, for example, be used during sports medical screening: most professional athletes already undergo X-rays of the knee in the medical screening process.

The results of our study are consistent with studies in the past, which have also found the notch width index and femoral notch size to be related to ACL rupture [26]. However, these previously conducted studies were primarily focused on anterior–posterior X-rays, while we used the Rosenberg view X-rays. The study of van Diek et al. [27] found no differences in morphology between patients with an ACL rupture and a control group in measurements with MRI. However, another MRI study performed by Whitney et al. [28] found a decreased femoral notch width to be related to ACL rupture. This was also a case-control study. A smaller femoral notch and smaller tibial eminence are related to smaller ACL size [29,30]. It is plausible that a smaller ACL could be less strong compared to a larger-sized ACL. The ACL is the main structure to prevent the bony relatively unstable lateral compartment from rotatory dislocation, i.e., rotation anterior of the tibia relative to the femur. The finding of a lower lateral tibia plateau in ACL patients could inspire the theory that these patients have even worse bony stability regarding the lateral compartment, which could be a risk factor for ACL injury.

In the lateral view X-rays, we did not find an association between shape variants and ACL rupture. Earlier, it has been demonstrated that the femoral condyle configuration [31] and the posterior tibial slope (PTS) [32,33,34] are related to increased stress on the ACL, but it is not known if this is connected to a higher risk of ACL rupture.

With the results of this study, we can identify individuals with certain shape variants of the knee, who are at greater risk for sustaining an ACL rupture. Because we did not use a predefined hypothesis, the found that risk factors are truly objective, whereas other researchers used a predefined hypothesis, potentially excluding numerous risk factors. These results can be used in daily practice, without the use of our program. Almost all clinicians can view the X-rays of their patients, and thus view the shape of the tibial eminence and the shape of the intercondylar notch.

Screening programs for professional athletes could focus on the intercondylar notch and tibial eminence as risk factors. With our results, screening programs could focus on the shape variants found and include patients with a higher risk of sustaining an ACL in their training programs, potentially making them more effective. In the past, research stated that the tibial slope could also be a determinant for sustaining an ACL rupture. With our hypothesis-free method, we did not find similar results. Excluding potential risk factors is also important because research should focus on risk factors that are more likely to be associated with sustaining ACL rupture

Although the odds ratios are relatively small, all the provided variants show a significant relationship to an ACL rupture, with odds ratios comparable to that of other studies investigating anatomical variants of the knee [16,27]. Further research could focus primarily on the shape variants found in this study and determine if these results can be reproduced. Furthermore, prospective studies should be performed to see if, with these risk factors, the prevalence of ACL ruptures could be reduced.

We understand that in the current literature there are already studies using 3D reconstructions of MRIs of the knee. However, our goal was not to confirm previously conducted research but to objectively describe the shape variants of the knee that contribute to the risk for sustaining an ACL rupture. If we had used MRI, we would have had to make a prior hypothesis. Furthermore, 3D reconstructions of the knee are not used in daily practice by every clinician. With the use of X-rays, we are confident that more clinicians can use these results in their practice, without the use of complicated extra software.

A drawback of SSM is that the shape represented by each variant needs to be reviewed personally (which we did in the consensus meeting). SSM does not provide a measurable cut off point; this should be determined in follow-up studies.

When we examined the different variants in our consensus meeting, we viewed 3D, moving animations of the shape variants; in this animation, the differences are more clearly visible than in 2D images.

We did not perform a power analysis before conducting the study, because we did not know how many shape variants would be found beforehand. Therefore, we used the Bonferroni correction for multiple testing. Although we understand that these are not the same thing, we are confident the method provided is valid and reproducible.

The strength of our study is the use of a large study population of 336 patients, who all practiced sports. Among the advantages of SSM is that the programs scale all differences in the size of the joints, thus reducing the variation in magnification and reducing measurement errors. A limitation of our study is the use of older control patients. We used older control patients to ensure that they were sufficiently exposed to rotational trauma. Older patients potentially have more degenerative changes. To ensure that this did not affect our outcomes, we chose patients with K&L scores of <1 (no signs of osteoarthritis) for both the patients with an ACL rupture and the control patients. Another limitation could be the use of a hypothesis-free program, which makes it, for some clinicians, harder to use in daily practice. However, we are confident that the shape variants found are usable in daily practice. You do not need a program to assess the wideness of the intercondylar notch in a Rosenberg view X-ray. Clinicians and radiologists with some experience in knee X-rays can easily interpret the findings in our study.

We used Rosenberg and standard lateral view X-rays for our analyses. In 1997, Shelbourne et al. [35] advocated the use of Rosenberg view X-rays because of the standardized protocol. The advantage of the use of X-rays is that they are easily obtained, relatively cheap, have a low patient radiation dose, and thus are ideal for identifying risk factors for sustaining an ACL rupture in large groups of asymptomatic patients.

An interesting sequel of this research would be to compare the differences in bony morphology between patients with and without a re-rupture after ACL reconstruction. This could help the clinician in giving the patient individualized information on the risk of re-rupture.

## 5. Conclusions

This study indicates that a smaller, flatter intercondylar notch; a lower lateral tibia plateau; a lower medial spike of the eminence; and a smaller tibial eminence were more common in patients who ruptured their ACL compared to control patients.

Further research should focus on ways to implement these differences in bony morphology in prevention programs to prevent ACL rupture in an individual who is at greater risk for sustaining ACL rupture.

## Figures and Tables

**Table 1:** Baseline demographic variables.

	ACL Injured (*n* = 168)	Control Group (*n* = 168)
Age, year	31 ± 7.4	38 ± 12.0
BMI, kg/m^2^	24.5 ± 3.4	24.7 ± 3.2
Female *n* (%)	49 (29.1)	49 (29.1)
Mean time in months between trauma and radiograph	1.0	6.9
Alternative/additional diagnosis, *n* (%)		
Medial Meniscus tear	10 (6)	57 (33.9)
Lateral meniscus tear	12 (7)	32 (19)
Cartilage lesion	60 (35)	15 (8.9)
Bone contusion	50 (30)	11 (6.5)
Collateral ligament lesion	0 (0)	7 (4.2)
No intra-articular lesions	0 (0)	46 (27.4)

Data are expressed as mean ± standard deviation or as *n* (%). BMI, body mass index. Anterior Cruciate Ligament (ACL)

**Table 2:** Shape variants associated with ACL rupture.

	Odds Ratio	95% C.I.	Sig.
Variant 1	2.2	(1.7–2.8)	0.001
Variant 3	1.8	(1.4–2.3)	0.001
Variant 6	2.1	(1.6–2.7)	0.001
Variant 10	1.5	(1.2–1.8)	0.001
Variant 17	1.4	(1.1–1.8)	0.0015

## Data Availability

The data presented in this study are available on request from the corresponding author. The data are not publicly available due to patients data privacy.
